# Laboratory Study on Disconnection Events in Comets

**DOI:** 10.1038/s41598-017-18712-0

**Published:** 2018-01-11

**Authors:** Yan-Fei Li, Yu-Tong Li, Wei-Min Wang, Da-Wei Yuan, Bao-Jun Zhu, Jia-Yong Zhong, Hui-Gang Wei, Fang Li, Bo Han, Kai Zhang, Xiao-Xing Pei, Zhe Zhang, Jia-Rui Zhao, Chang Liu, Guo-Qian Liao, Zhi-Heng Fang, Chen Wang, Xiao-Gang Wang, Youichi Sakawa, Yong-Joo Rhee, Xin Lu, Neng Hua, Bao-Qiang Zhu, Taichi Morita, Yasuhiro Kuramitsu, Xiu-Guang Huang, Si-Zu Fu, Jian-Qiang Zhu, Gang Zhao, Jie Zhang

**Affiliations:** 1 Beijing National Laboratory for Condensed Matter Physics, Institute of Physics, Chinese Academy of Sciences, Beijing, 100190 China; 2 Key Laboratory of Optical Astronomy, National Astronomical Observatories, Chinese Academy of Sciences, Beijing, 100012 China; 3 Department of Astronomy, Beijing Normal University, Beijing, 100875 China; 4 Shanghai Institute of Laser Plasma, Chinese Academy of Engineering Physics, Shanghai, 201800 China; 5 Department of Physics, Harbin Institute of Technology, Harbin, 150001 China; 6 Institute of Laser Engineering, Osaka University, 2-6 Yamadaoka, Suita, Osaka, 565-0871 Japan; 7 Center for Relativistic Laser Science, Institute for Basic Science, Gwangju, 61005 Korea; 8 National Laboratory on High Power Lasers and Physics,Chinese Academy of Sciences, Shanghai, 201800 China; 9 Interdisciplinary Graduate School of Engineering Sciences, Kyushu University, Kasuga, Fukuoka, 816-8580 Japan; 10 Department of Physics, National Central University, Jung-Li, 32001 Taiwan; 11 Key Laboratory for Laser Plasmas (MoE) and Department of Physics and Astronomy, Shanghai Jiao Tong University, Shanghai, 200240 China; 12 Collaborative Innovation Centre of IFSA (CICIFSA), Shanghai Jiao Tong University, Shanghai, 200240 China; 13 School of Physical Sciences, University of Chinese Academy of Sciences, Beijing, 100049 China

## Abstract

When comets interacting with solar wind, straight and narrow plasma tails will be often formed. The most remarkable phenomenon of the plasma tails is the disconnection event, in which a plasma tail is uprooted from the comet’s head and moves away from the comet. In this paper, the interaction process between a comet and solar wind is simulated by using a laser-driven plasma cloud to hit a cylinder obstacle. A disconnected plasma tail is observed behind the obstacle by optical shadowgraphy and interferometry. Our particle-in-cell simulations show that the difference in thermal velocity between ions and electrons induces an electrostatic field behind the obstacle. This field can lead to the convergence of ions to the central region, resulting in a disconnected plasma tail. This electrostatic-field-induced model may be a possible explanation for the disconnection events of cometary tails.

## Introduction

The cometary nucleus is a single solid body of icy conglomerate, composed of a mixture of frozen gases and stony meteoritic materials^1^. When approaching the Sun, comets would be heated and their ices start to sublimate under the intense solar radiation, leading to the formation of comas. In general, the diameter of a cometary nucleus can vary from 100 m to more than 40 km, while the diameter of a coma can reach thousand kilometers. The nucleus and the coma will continue to transform to become long cometary tails^2^. There are two types of cometary tails, dust tail and plasma tail^3^. The dust tail, which can be more than one, is spread out over a wide region. Influenced mainly from the orbital path of the comet, the dust tail appears curved. While the plasma tail is typically straight and narrow, lying along the sun-comet line, due to being shaped by both the solar wind flow field and the interplanetary magnetic field (IMF). The most remarkable phenomenon that occurs in the plasma tail is the disconnection event (DE) in which the plasma tail is uprooted from the comet’s head and moves away from the comet. Theories explaining the onset of DEs can be grouped into three classes based on the triggering mechanisms, namely ion production effects, pressure effects and magnetic reconnection. However, just the latter two theories are believed to be reasonable currently. Pressure effects theory, first put forward by IP and Mendis, is described as when the dynamic pressure of the solar wind increases considerably, the comet’s ionosphere would be compressed and the magnetic field lines would be changed or various instabilities, such as Rayleigh-Taylor instability, would be excited in the tail, then a DE happens^4^. However, a recent work reveals that the DE onsets of comet P/Halley correlated with pressure effects are only in 23% of the analyzed cases^5^.

There are two models for the magnetic-reconnection theory^6–9^. In 1978, Niedner and Brandt first proposed that when a comet crossed the IMF sector boundary, i.e., the heliospheric neutral sheet (HCS), the sunward magnetic reconnection occurred^6^. Consequently the plasma was uprooted and moved away from the reconnection region. This model was not corroborated until 2007 by Jia *et al*.’s simulated results obtained with a time-dependent, fully three-dimensional self-consistent ideal magnetohydrodynamic (MHD) model^7^. However, after analyzed the observed data from the Vega satellite, Delva *et al*. found that about half of the DEs related to the HCS crossing was plausible. Furthermore, HCS crossing was neither a necessary nor a sufficient condition for a DE^10^. Different from the sunward magnetic reconnection model mentioned above, Rusell *et al*. proposed another tailside reconnection model, which might be triggered by an interplanetary corotating shock or a high-speed stream^8^. However, the simulated and observed results show that some DEs cannot be caused by the tailside reconnection model either^9,11^. From the above discussions we can see further work is needed to understand the DE triggering mechanisms.

Recent years high-power laser-plasma experiments provide opportunities to study astrophysics^12–23^. With the similarity criteria^24–26^, which can scale the laboratory systems to the astrophysical ones, many laser-driven experiments have been performed to understand astrophysical problems^23,27–33^. In this paper, the interaction process between solar wind and a comet is simulated with a laser-driven plasma cloud colliding with a cylinder obstacle. A disconnected plasma tail behind the obstacle is observed by optical measurements. Our particle-in-cell simulations show that the difference in thermal velocity between ions and electrons will induce an electrostatic field behind the obstacle. This field leads to the convergence of ions, and the disconnected plasma tail. This process may be a possible explanation for the disconnection events of a comet.

## Experiment results

**Figure 1:**
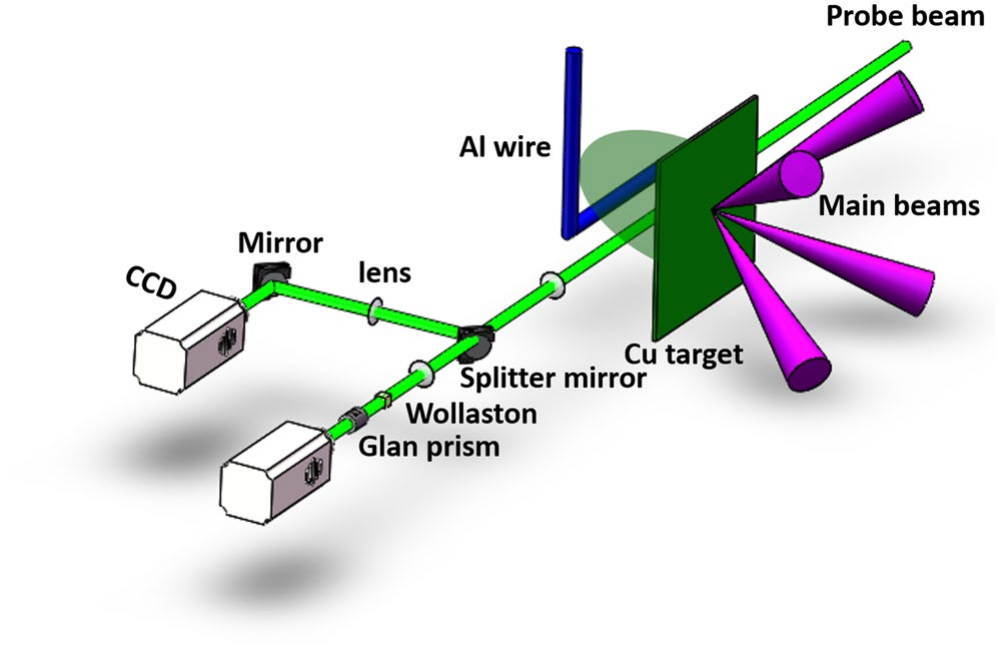
Schematic view of the experimental setup. Four 240 J, 1 ns, 0.351 μm laser beams were incident on the front surface of a 2 × 2 × 0.006 mm^3^ Cu planar target. The forward supersonic plasma produced at the rear of the target interacts with an aluminum wire placed 1 mm away from the Cu target. The interaction was measured by shadowgraphy and Nomarski interferometry with a 527 nm, 30 ps short laser probe.

The experiments were carried out on the Shenguang II (SG II) laser facility at the National Laboratory on High Power Lasers and Physics. The experimental setup and target configuration are schematically shown in Fig. 1 and more details are shown in the Methods.

**Figure 2:**
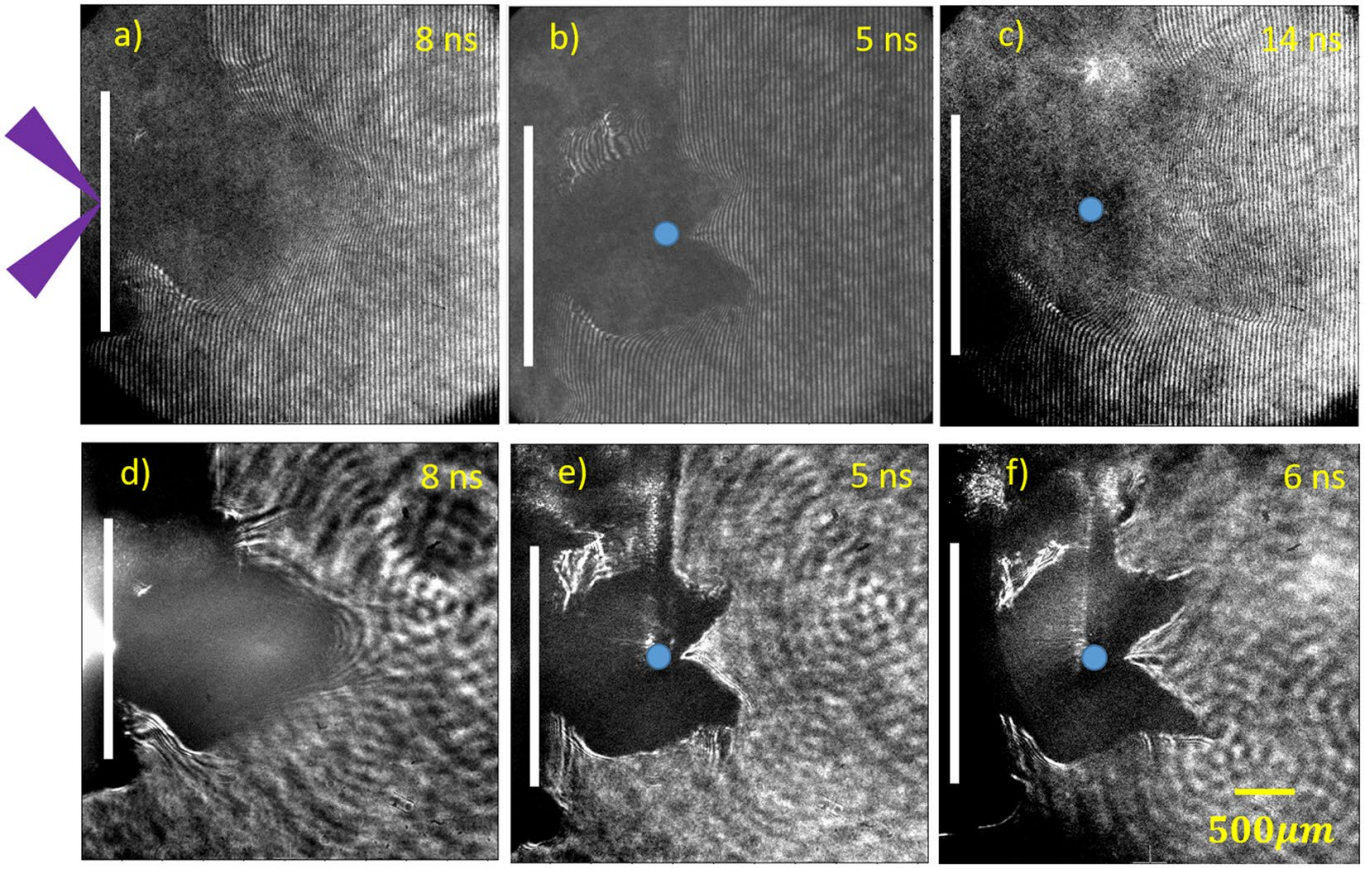
(**a**) Interferogram and (**d**) shadowgraph of the plasma cloud without the obstacle, taken at a delay time of 8 ns. (**b**) and (**c**) are the interferograms with the obstacle taken at 5 ns and 14 ns, respectively. (**e**) and (**f**) are the shadowgraphs with the obstacle taken at 5 ns and 6 ns, respectively. The purple arrows represent the main laser beams. The white lines indicate the original positions of the Cu planar target. The blue solid circles indicate the cross section of the obstacle.

Figure 2 shows the observed interferograms and shadowgraphs. The original target foils are marked by the white lines. The blue solid circles indicate the cross section of the cylinder obstacle. The purple arrows represent the main laser beams. After the main laser irradiation, a supersonic plasma cloud ejected from the rear-side of the Cu target to the right is produced. We firstly characterized this forward plasma cloud without the obstacle. The typical interferogram and shadowgraph of the plasma cloud taken at 8 ns are shown in Fig. 2(a) and 2(d), respectively. The dark regions in the pictures correspond to the high-density or large-density gradient regions, where the probe light is absorbed or refracted out of the imaging optical system. With the Abel inversion, the local electron density of the plasma cloud, *n*_e_, at the detectable boundary is ~10^19^ cm^−3^. The boundary reaches 2.3 mm away from the initial target surface at 8 ns, indicating an average expending speed of ~280 km/s.

Figure 2(b) and 2(e) show the interferogram and shadowgraph with the obstacle taken at 5 ns, respectively. After colliding with the obstacle, the plasma cloud is split into two parts. The most striking feature is presence of a tiny plasma tail behind the obstacle in the axial direction of the plasma cloud. Moreover, the tail is disconnected from the plasma cloud and the obstacle. Figure 2(f) and 2(c) show the shadowgraph taken at 6 ns and interferogram at 14 ns, respectively. Compared with that at 5 ns, the disconnection distance between the tail and the plasma cloud is increased with time. We estimate the speed of the disconnection point moving away to be ~100 km/s.

## Simulation results

The collimation and disconnection features of the generated plasma tail are very similar to those of the cometary plasma tails related to DEs. To understand the generation of the disconnected plasma tail, we have performed two-dimension (2D) particle-in-cell (PIC) simulations to observe the evolution of the plasma cloud by the KLAPS code^34^. The whole process should consist of three phases, the generation of the forward plasma cloud, collision of the cloud with the obstacle, and the evolution of the two split-plasma bunches. It is difficult to include the whole process with a timescale of tens ns in PIC simulations, due to numerical noise and computational time. Therefore, we only simulate the evolution of the two split-plasma bunches just behind the obstacle, which directly correlates with the tail disconnection.

**Figure 3:**
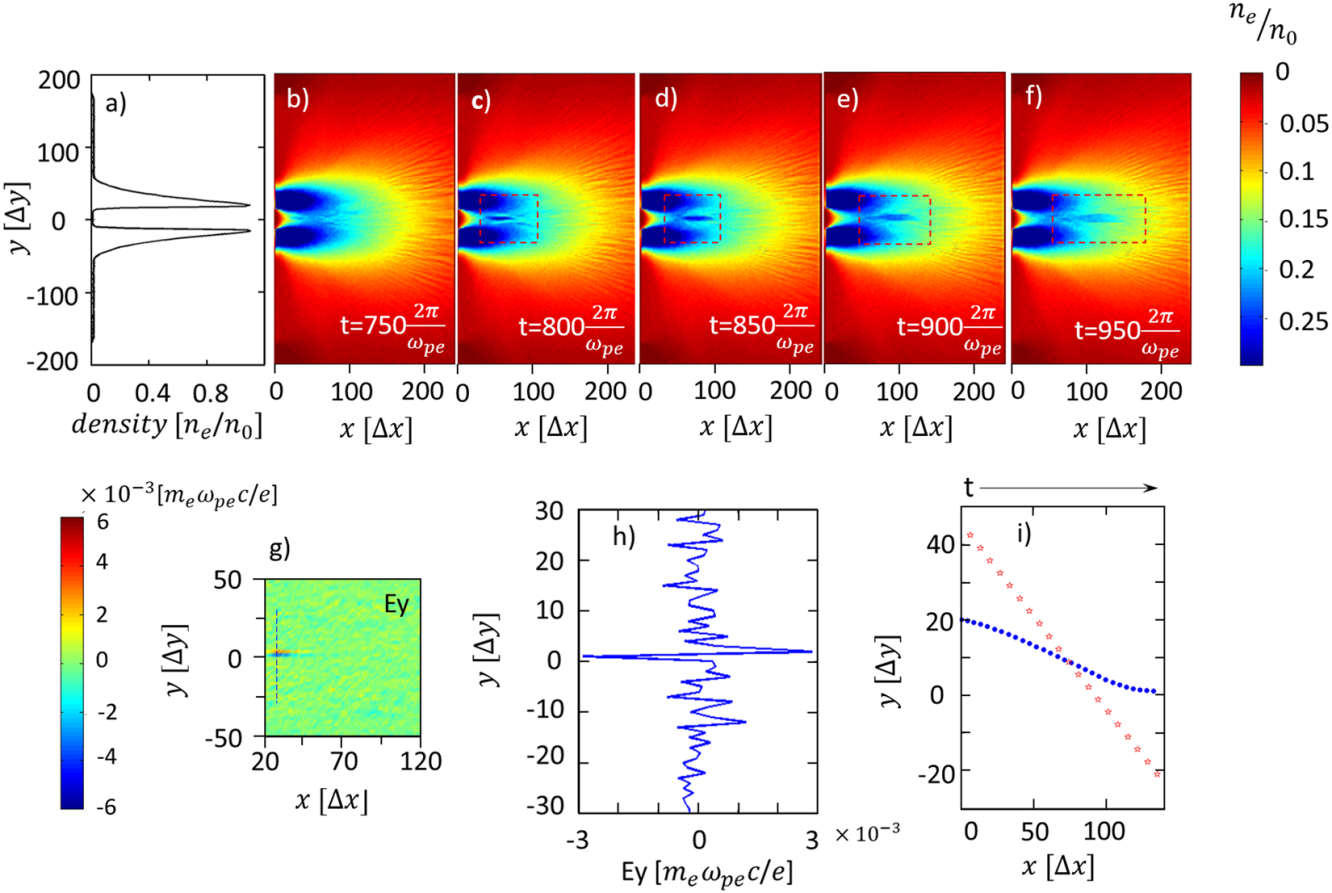
(**a**) The initial density profile of the plasma cloud in the *y* direction at *x* = 0, used in the simulations. (**b**–**f**) the electron density distributions in the *x*-*y* plane at different times. (**g**) The distribution of the electrostatic field *E*_*y*_ at ${\rm{t}}=750\frac{2{\boldsymbol{\pi }}}{{{\boldsymbol{\omega }}}_{{\boldsymbol{pe}}}}$. (**h**) The lineout of *E*_*y*_ along the dashed line in (**g**). (**i**) Typical traces of an ion trapped in the tail (blue point) and an ion going through the central region (red star) with time going on.

Figure 3 shows the simulation results, where Fig. 3(a) shows the initial electron density profile (*n*_e_/*n*_0_) of the plasma cloud at the source (*x* = 0 position), in which the density in the middle region is ~0, and Fig. 3(b–f) show the temporal evolution of electron density distributions in *x-y* space with time. Although the plasma cloud moves along the +x direction at initial time, the upper and lower plasma bunches reach the middle region at ${\rm{t}}=750\frac{2\pi }{{\omega }_{pe}}$ because of the transverse thermal expansion. At ${\rm{t}}=800\frac{2\pi }{{\omega }_{pe}}$, a disconnected plasma tail is obviously formed in the middle region. From Fig. 3(c–f), we can see that the tail is moving to the right. The density of tail becomes as high as *n*_e_ ~ 0.28 *n*_0_ at ${\rm{t}}=850\frac{2\pi }{{\omega }_{pe}}$. At ${\rm{t}}=950\frac{2\pi }{{\omega }_{pe}}$ it starts to dissipate. The disconnected tail in the simulation is very similar to the experimental results in Fig. 2.

In our simulations we find an electrostatic (*E*) field generated between the two plasma bunches. Fig. 3(g–h) show the spatial distribution and lineout of *E*_*y*_ at ${\rm{t}}=750\frac{2\pi }{{\omega }_{pe}}$. We can see that *E*_*y*_ appears bipolar in the middle region. The *E* field originates from the difference in electron and ion mass. At the beginning, the plasma electrons and ions have the same temperature. The thermal speed of the electrons is much higher than that of the ions. The electrons move fast and the ions follow them behind. Thus the middle region is filled with electrons firstly. Attracted by the electrons, more and more ions fill in the region. The accumulation of the ions triggers an *E* field. Under the influence of *E* field, the electrons will be pulled back and vibrate around ions. With more ions accumulated in the middle region, the bipolar *E* field shown in Fig. 3 (g) is formed.

The *E* field will affect the ion and electron dynamics greatly. An ion from the upper region expanding in −*y* direction will be decelerated by the positive *E* field in the upper half region firstly. If its *v*_*y*_ is high enough to go through the upper *E* field region, it will go into the negative *E* field at the lower part and be accelerated away from the middle region. Its trace evolving with time is shown with the red stars in Fig. 3(i). However, if the ion speed is not so high, it will be decelerated by the positive *E* field and trapped in the middle region. A typical trace of such a trapped ion is also shown with the blue points in Fig. 3(i). This process is also true for an ion moving in the +*y* direction from the lower region. With more and more low or medium speed ions trapped, a plasma tail with disconnected structure is gradually formed and moving with the *E* field.

To further examine the idea that the disconnected tail is formed because the velocity difference between the electrons and ions, we also perform an additional simulation with higher temperature of ions and do not observe a clear disconnected tail formed.

Both in experiment and simulation the plasma tail is disconnected from the obstacle and moves away, behaving like the disconnected cometary plasma tail in a DE. This is because the *E* field is induced at a distance away from the *x* = 0 position (obstacle position) and drifting to the right. Therefore the *E* field induced plasma tail is disconnected from the obstacle and also moves away. With more ions accumulated, the tail tends to diffuse. It can be seen from Fig. 3(c–f), at ${\rm{t}}=800\frac{2\pi }{{\omega }_{pe}}$ the width of plasma tail is about 8Δy, while at ${\rm{t}}=950\frac{2\pi }{{\omega }_{pe}}$ it becomes about 12Δ*y*. The diffusion velocity is about 0.0007*c*_*L*_, which is far less than the initial thermal velocity of ions, 0.01*c*_*L*_. This is an evidence that the ions are confined by the *E* field in the *y*-direction in the tail region.

Note that the evolution time of the simulated tail is $\sim 200\frac{2\pi }{{\omega }_{pe}}$, which corresponds to $\sim 30\frac{2\pi }{{\omega }_{pi}}$, where *ω*_*pi*_ is the ion plasma frequency. This evolution time is much lower than the experimental one $( \sim {10}^{4}\frac{2\pi }{{\omega }_{pi}})$. This is reasonable since the spatial scale is reduced by 30 times and the speed of plasma is increased by 30 times in the simulations due to the down-scaled light speed, ${c}_{L}=\frac{1}{30}{c}_{real}$.

## Discussions

The solar wind plasma is typically magnetized, whose evolution is described by MHD models. However, it should be noted that the size of most comets are shorter than the cyclotron radius of ions in solar wind, which is about 10^3^ km^35^. In this case, one could pay more attention to the interplays among charged particles in DE processes. This has been verified by our experiments and simulations without a magnetic field included. Our results show that the interplays among charged particles can induce the generation of an electrostatic field when the density of plasma cloud is high, this electrostatic field can cause the convergence of ions of the tail plasma, and that the converged ions move with the electrostatic field away from the obstacle, leading to the appearance of a disconnected plasma tail. Correspondingly, when the density of solar wind rises, the process similar to the experimental process happens, triggering a DE.

With the similarity criteria^24^, we have created the system in the laboratory which can be scaled to the astrophysical one to simulate the process of solar wind interacting with a comet. However, The Reynolds number, Re, and the Peclet number, Pe, are required to be $\mathrm{Re}=\frac{hv}{\gamma }\gg 1$ and ${\rm{Pe}}=\frac{hv}{\chi }\gg 1$, where *h* is characteristic length and taken as the diameter of the comet/obstacle here, *v* is the speed of solar wind/plasma cloud, *γ* and *χ* are the kinematic viscosity and the thermal diffusivity, respectively, to ensure that the viscosity and heat conduction in the laboratory and in the astrophysical system are unimportant. For the solar wind, $\gamma =2\times {10}^{13}c{m}^{2}{s}^{-1},\frac{hv}{\gamma }\approx 2\times {10}^{5}\gg 1$ and $\chi  > 8.6\times {10}^{14}c{m}^{2}{s}^{-1},\frac{hv}{\chi } > 5\times {10}^{3}\gg 1$. As for the experimental plasma cloud, $\gamma =0.25\,c{m}^{2}{s}^{-1},\frac{hv}{\gamma }\approx$ $2.24\times {10}^{6}\gg 1$ and ${\rm{\chi }}\approx 2\times {10}^{4}\,c{m}^{2}{s}^{-1},\frac{hv}{\chi }\approx 28\gg 1$ ^24^. In order to satisfy the behavior as a fluid, particles in the plasma should be localized, i.e.,$\,\frac{{r}_{Li}}{h}\ll 1$ to the solar wind case, and $\frac{{l}_{c}}{h}\ll 1$ to the experimental plasma cloud case, where *r*_*Li*_ is the ion Larmor radius and *l*_*c*_ is the collisional mean free path. To the solar wind, ${r}_{Li}\approx {10}^{3}\,km$. The diameters of comets vary in a large range. For example, the diameter of P/Halley’s coma is up to 10^6^ km, while many other ones cannot reach 10^3^ km. However, in the interaction region, in front of a comet, the compressed plasma is much denser, resulting in a smaller *r*_*Li*_ thus, $\frac{{r}_{Li}}{h}\ll 1$, generally. As for the undisturbed experimental plasma cloud, $h\approx 200\,\mu m,\frac{{l}_{c}}{h}\approx 3\times {10}^{13}\frac{{T}^{2}}{{\rm{\Lambda }}{n}_{i}h}\approx 0.15$. In the interaction region $\frac{{l}_{c}}{h} < 0.15\ll 1$. The similarity between the experiment and astrophysical process is determined by the Euler number, ${E}_{u}=v{(\rho /p)}^{1/2}$. However, as the dynamic pressure is dominated by $p\sim \rho {v}^{2}$, the Euler numbers are the same here. Therefore, the interaction between the plasma cloud and the cylinder obstacle can be scaled to the interaction between solar wind and a comet. Some parameters are list as Table 1. It can be seen that the diameter of the comet which can be simulated here is about 10^3^ km. These comets are named weak comets.

**Table 1:** Characteristic parameters of laser-produced plasmas, simulated plasma cloud and solar wind.

parameters	experiment	PIC simulation	DEs
*A*	64	1	1
*Z*	10	1	1
*T*	100 eV	${10}^{-4}{m}_{i}{{c}_{L}}^{2}$	10 eV
*n*_*i*_	10^19^ cm^−3^	10^19^ cm^−3^	1 cm^−3^
*B*	—	—	10 nT
*d*_*i*_	54 μm	20Δy	2.29 × 10^2^ km
*d*_*e*_	0.5 μm	3Δy	5.32 km
*d*	200 μm	20Δy	—
*c*_*s*_	41 km/s	0.01*c*_*L*_	45 km/s
*v*_*A*_	—	—	220 km/s
*v*	280 km/s	0.02*c*_*L*_	300–700 km/s
Mach-number, *M*	7	2	6.7–15.5
*M*_*A*_	—	—	1.6–3.2

where *A* is atomic weight, *Z* is the average ionization state, *n*_*i*_ is ion density of plasma cloud or solar wind, *B* is magnetic flux density, *d*_*i*_ is ion inertial length, *d*_*e*_ is plasma skin depth, *d* is the diameter of obstacle or comet, *c*_*s*_ is ion sound velocity, *v*_*A*_ is Alfven velocity, *v* is velocity, *M*_*A*_ is Alfven Mach-number, *c*_*L*_ is the speed of light in simulations, ${\rm{\Delta }}{\rm{y}}=0.05\frac{2\pi {c}_{L}}{{\omega }_{pe}}$ is the cell size.

In addition, after the interaction, the density of the plasma just behind the obstacle is low. The electron density can be calculated as *n*_*e*_ ~ 10^19^ cm^−3^ from the interferogram with the Abel inversion. Thus *n*_*i*_ ~10^18^ cm^−3^, *l*_*c*_ ≈ 300 μm and *d*_*i*_ ≈ 170 μm here. The width of the plasma tail is ~50 μm, which is smaller than *l*_*c*_ and *d*_*i*_. Therefore, it is reasonable to simulate the evolving process with PIC code after the interaction.

## Conclusions

In conclusion, the interaction between solar wind and comets is simulated by means of laser-driven plasma cloud colliding with a cylinder obstacle. A disconnected plasma tail is observed by shadowgraphy and interferometry. Particle-in-cell simulations show that the difference in thermal velocity between ions and electrons causes an electrostatic field behind the obstacle, which leads to the convergence of ions and a disconnected plasma tail. This provides another possible explanation for the disconnection events of comets, besides the mechanisms proposed previously.

## Methods

### Experiment setup

Four 240 J, 1 ns, 351 nm laser beams were incident on the front surface of a 2 × 2 × 0.006 mm^3^ Cu planar foil to produce a forward supersonic plasma at the rear side of the target. In order to simulate the solar wind, a plasma with a large transverse size and a high longitudinal speed is necessary. This requires the diameter of the laser focal spot should be large enough but also keep the laser intensity high. To do this, we set the diameter of the laser focal spot on the target surface to be ~600 µm, which gave an average intensity of 3.4 × 10^14^ W/ cm^2^. A ∅ 200 μm L-shape Al wire was placed 1 mm away from the Cu target. The horizontal part of the wire acted as a 2-dimenstional comet-like obstacle. The vertical part of the wire was the holder. The axis of the horizontal part of the wire was parallel to the Cu target plane and at the same height as the Cu target center, where the main laser beams hit. A 527 nm laser beam with a short duration of 30 ps, was used as an optical probe. The propagation direction of the probe beam was aligned in parallel with axis of the cylinder obstacle. Shadwgraphy and Nomarski interferometry, with a magnification factor ~3, were used to measure the spatial and temporal evolution of the interaction. A time series of snapshots were obtained by changing the delay between the probe and the main beams. The delay time was defined as the time separation between the falling edges of the probe and the main beams.

### PIC Simulations

Owing to the limitation of the tremendous computational time, in our simulations a down-scaled ratio of the ion and electron mass, $\frac{{m}_{i}}{{m}_{e}}=40$, and a low light speed, ${c}_{L}=\frac{1}{30}{c}_{real}$, are used, where *m*_*i*_ and *m*_*e*_ are the ion mass and electron mass, respectively, and *c*_*real*_ is the real light speed in vacuum. This method has been also applied in previous simulations^36–38^.

To describe a plasma cloud with two split bunches, the initial electron density profile (*n*_e_/*n*_0_) of the plasma cloud at the source (*x* = 0 position) is set as a Gaussian distribution, in which the density in the middle region is ~0, as shown in Fig. 3(a). The plasma cloud is injected from the x = 0 position into the simulation box with a speed of 0.02 *c*_*L*_ (≈200 km/s). The initial electron and ion densities are the same (*Z* = 1). The initial electron and ion temperatures are set as $\,{T}_{i}={T}_{e}=0.004\,{m}_{e}{c}_{L}^{2}={10}^{-4}{m}_{i}{c}_{L}^{2}$, which is close to the ones, about 100 eV, observed in the previous experiments^39,40^. The spatial resolution or the cell size is taken as ${\rm{\Delta }}{\rm{x}}={\rm{\Delta }}{\rm{y}}=0.05\frac{2\pi {c}_{L}}{{\omega }_{pe}}$, and the temporal resolution is ${\rm{\Delta }}{\rm{t}}=0.025\frac{2\pi }{{\omega }_{pe}}$, where *ω*_*pe*_ is the electron plasma frequency. The simulation box is set as *N*_*x*_ × *N*_*y*_ = 300Δx × 400Δy. The left boundary is at x = −60 Δx position which is not shown in Fig. 3. Absorption boundary conditions are adopted in both *x* and *y* directions. 1000 simulation particles are assigned per cell for both electrons and ions.

### Data availability statement

The authors declare data in the manuscript is of availability.
